# Low-Cost and Scalable Platform with Multiplexed Microwell Array Biochip for Rapid Diagnosis of COVID-19

**DOI:** 10.34133/2021/2813643

**Published:** 2021-03-12

**Authors:** Yang Wang, Kaiju Li, Gaolian Xu, Chuan Chen, Guiqin Song, Zaizai Dong, Long Lin, Yu Wang, Zhiyong Xu, Mingxia Yu, Xinge Yu, Binwu Ying, Yubo Fan, Lingqian Chang, Jia Geng

**Affiliations:** ^1^Beijing Advanced Innovation Center for Biomedical Engineering, Key Laboratory for Biomechanics and Mechanobiology, School of Biological Science and Medical Engineering, Beihang University, Beijing 100083, China; ^2^Department of Laboratory Medicine, State Key Laboratory of Biotherapy and Cancer Center, West China Hospital, Sichuan University and Collaborative Innovation Center, Chengdu 610041, China; ^3^Nano Biomedical Research Centre, School of Biomedical Engineering, Shanghai Jiao Tong University, Shanghai 200030, China; ^4^Wuhan Chain Medical Labs, Wuhan, Hubei 430011, China; ^5^Department of Clinical Laboratory, Zhongnan Hospital of Wuhan University, Wuhan, Hubei 430071, China; ^6^Department of Biomedical Engineering, City University of Hong Kong, China

## Abstract

Sensitive detection of SARS-CoV-2 is of great importance for inhibiting the current pandemic of COVID-19. Here, we report a simple yet efficient platform integrating a portable and low-cost custom-made detector and a novel microwell array biochip for rapid and accurate detection of SARS-CoV-2. The instrument exhibits expedited amplification speed that enables colorimetric read-out within 25 minutes. A polymeric chip with a laser-engraved microwell array was developed to process the reaction between the primers and the respiratory swab RNA extracts, based on reverse transcriptase loop-mediated isothermal amplification (RT-LAMP). To achieve clinically acceptable performance, we synthesized a group of six primers to identify the conserved regions of the ORF1ab gene of SARS-CoV-2. Clinical trials were conducted with 87 PCR-positive and 43 PCR-negative patient samples. The platform demonstrated both high sensitivity (95.40%) and high specificity (95.35%), showing potentials for rapid and user-friendly diagnosis of COVID-19 among many other infectious pathogens.

## 1. Introduction

Coronavirus disease 2019 (COVID-19) has been a life-threatening pandemic caused by SARS-CoV-2 [1, 2]. It consists of four structural proteins and a single-stranded RNA as the genetic materials [3]. The rapid transmission of the virus among human beings has caused wide threats to the public health [4–8]. Up to August 23, 2020, the disease has spread to more than 200 countries, infected over 23.2 million people, and resulted in at least 800,000 deaths [9]. There is an urgent need on techniques for rapid and accurate detection of SARS-CoV-2 to inhibit its further spreading. Point-of-care testing (POCT) platforms with advantages of low-cost, easy operation, and high sensitivity/specificity are broadly applicable to daily life [10, 11].

The symptoms of the patients with the infection of COVID-19 are nonspecific, so various diagnostic methods have been developed accordingly [12–17]. To rapidly screen the subject infected with COVID-19, nucleic acid testing (NAT) based on the reverse transcription-quantitative polymerase chain reaction (RT-qPCR) is now applied as a clinical-acceptable standard [18, 19]. Till now, RT-qPCR has been proved to be highly sensitive and specific [20, 21]. However, its dependence on expensive thermocycler and read-out systems limits the application, especially in some scenarios where the exponential increase of the infected patients exceeds the capacity of PCR instruments during the early outbreak of COVID-19 [22]. By contrast, isothermal amplification techniques conducted at fixed temperature do not rely on the expensive instruments for thermal cycling, thus offering the convenience for wide scopes, such as community hospitals, homes, or remote areas [23, 24]. Loop-mediated isothermal amplification (LAMP) is a low-cost yet rapid isothermal approach, which enables the identification of the target nucleic acid fragment of the virus within 1 hour, by using a set of primers and a strand-displacement polymerase at a constant temperature (60-65°C) [25–32]. To detect SARS-CoV-2, one type of RNA virus, reverse transcriptase (RT) is added in the LAMP system to initiate the amplification, known as RT-LAMP [33, 34]. The testing results can be visualized by adding a coupled pH indicator or a fluorescent dye in the reaction buffer [35, 36].

In this work, we developed a portable and scalable platform integrated with a multiplexed RT-LAMP microwell array biochip for rapid detection of SARS-CoV-2. The platform achieves full functions, including sample pretreatment, nucleic acid cleavage and enrichment, isothermal amplification, and colorimetric detection. On the platform, a simple laser-engraved microwell array chip was developed for multiplexed amplification of the viral RNA samples. We designed a group of six novel primers for specific identification of the conserved regions of the ORF1ab gene of SARS-CoV-2. To ascertain the rate of false results, both negative and positive controls were designed on the chip. A digital image sensing module and a liquid crystal display (LCD) were developed to detect the amplification results in each microwell. The platform achieved a low limit of detection of 1,000 copies/mL within 25 min for SARS-CoV-2 detection. Both high sensitivity (95.40%) and selectivity (95.35%) were achieved when applied this platform to test 130 clinical samples by double-blind testing at the West China Hospital, Chengdu, China. The overall performance of the platform proved its advantage as a portable and low-cost technique for rapid diagnosis of the infectious pathogens.

## 2. Results

### 2.1. Basic Concept of the Point-of-Care Detection Platform

**Figure 1:**
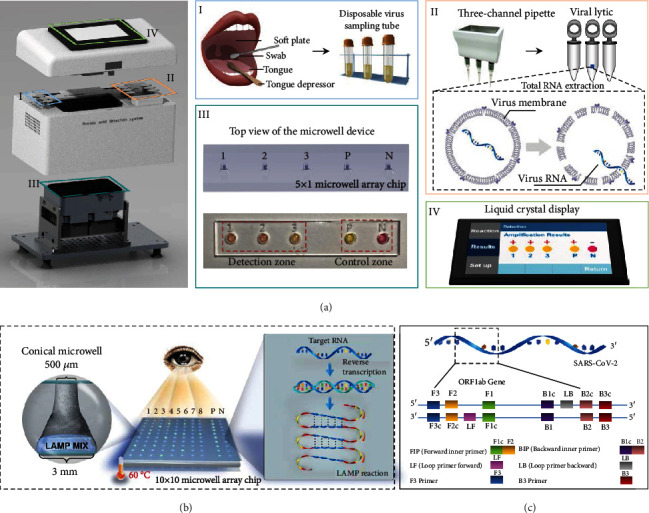
System setup of the portable instrument and multiplexed microwell array biochip based on RT-LAMP for point-of-care testing of SARS-CoV-2. (a) The designed portable detection instrument with labelled parts: (I) the area for loading human tissue sampling and preservation in a small sampling tube—patients collect their own tissue sample through pharyngeal swab; (II) the zone for total RNA extraction—preserved samples are transferred for inactivation and RNA extraction in virus lysis buffer using a custom-made three-channel pipette; (III) multiplexed microwell array biochip for RT-LAMP reaction—top view of the 5 × 1 microwell device with a detection zone and control zone, which detects three clinical samples. The BRAC1 gene is used as the positive control, and ddH_2_O is used as the negative control; (IV) display module of the detector—all detection results and parameter settings are integrated on the liquid crystal display of the instrument. (b) The schematic of a RT-LAMP biochip for simultaneous detection of multiple samples. For testing, RNA samples are added into the microwell prefilled with our designed LAMP solution. The amplification reaction, occurring at 60°C, leads to a colorimetric change in the solution that can be visualized by the digital image sensor or with the naked eye. The microwells for RNA amplification were engraved with a commercial laser system. The microwell designed in conical shape was demonstrated to speed up the amplification as compared to commercial PCR tubes. (c) The genome map shows the LAMP primers targeting at the ORF1ab gene of the SARS-CoV-2. The inner primers (FIP and BIP) consist of two distinct parts of target, F2+F1c and B2+B1c, respectively. The loop primers (LoopF and LoopB) are designed by using the complementary strands according to the fragments between F1 and F2, or B1c and B2c.

The setup of the platform and the microwell array-based RT-LAMP biochip is illustrated in Figure 1 (see Figure S1 for detailed circuit design and power delivery diagram). Four functional modules are designed and integrated on the platform, which implement the whole procedure including human throat swab sampling, RNA extraction, RT-LAMP amplification, and results reading (Figure 1(a), I to IV). During detection, the raw respiratory samples are collected with the aid of a swab and preserved in the sampling zone of the instrument, where the small tubes have been prefilled with virus preservation fluids. Subsequently, the collected samples are transferred with a three-channel pipette to the zone for viral inactivation and RNA extraction. The microwells prefilled with RT-LAMP buffer are applied to mix with the extracted RNAs, then initiate the isothermal amplification on the instrument under 60°C. Typically, a LAMP reaction experiences three steps, including “starting material producing step”, “cycling amplification step”, and “elongation and recycling step” [37]. A dumb-bell structure formed in the first step acts as the template for new DNA strand synthesis in the following two steps. In this work, for quick proof-of-concept, a polymethyl methacrylate (PMMA) chip with 5 × 1 microwells, including three for testing and two for control, was fabricated by laser engraving. Particularly, a conical-shaped microwell, with the top diameter of 500 *μ*m and the bottom diameter of 3 mm, was adopted and designed (Figure 1(b), S3). The two external-control microwells are used to ascertain the rates of false-positive and false-negative results, further normalizing the output signal intensity across the devices for quantitative analysis. To achieve rapid and easy readout, we applied a colorimetric pH indicator (phenol red) in the reaction buffer. Once the amplification occurs, the pH variations lead to a color change of the buffer from pink to yellow. The color shift is proportional to the concentration of the target RNA [28]. The diagnostic result (i.e., positive (P) or negative (N) for SARS-CoV-2) in every microwell is scanned by a digital image sensor and is shown on a liquid crystal display (LCD) on the instrument. Notably, such a color change can also be visualized by the naked eye. Parameters of RT-LAMP settings, including reaction time and temperature, are set on the LCD (Figure S2).

The LAMP primers for detecting SARS-CoV-2 were designed based on the open reading frame 1ab (ORF1ab) gene, which was recommended by the Chinese Center for Disease Control and Prevention (CDC) [38]. We designed six novel specific primers, termed as inner primers (BIP and FIP), outer primers (B3 and F3), and loop primers (LoopF and LoopB) (Figure 1(c), see Table S1 for detailed sequences). The primers have obvious influences on the precise size of the amplification product during the cycling stage.

### 2.2. Optimizations for the LAMP Assay

**Figure 2:**
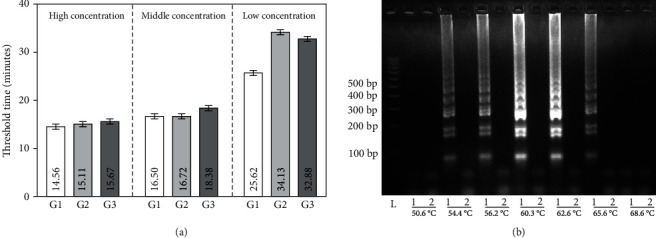
The optimization results for the diagnosis of SARS-CoV-2. (a) The threshold time as a function of the different groups of primers with different concentrations of targets. The targets in three different concentrations, including low concentration (10^7^ copies/mL), middle concentration (10^8^ copies/mL), and high concentration (10^9^ copies/mL) were recorded based on different sets of primers. The data are expressed as the mean ± SD (*n* = 3). (b) Temperatures ranging from 50°C to 68°C for the RT-LAMP reactions were optimized using 10^5^ copies/mL of targets with gel electrophoresis.

To investigate the properties of different sets of designed primers (G1-G3), real-time RT-LAMP reactions were carried out. The results of negative reactions indicate that no nonspecific amplification occurred in the reactions, even with long incubation time up to 40 mins (Figure S4). More importantly, the results with a 10-fold serially diluted target ranging from 10^7^ copies/mL to 10^9^ copies/mL indicate that all sets of primers can be used for amplification and detection of the SARS-CoV-2. Compared with the other two sets of primers (G2 and G3), the threshold time (defined as the time corresponding to 50% of the maximum fluorescence intensity, *T*_*t*_) needed for the nucleic acid amplification based on the first set of primer (G1) is relatively less, which means a faster reaction speed, especially for those targets with low concentrations (Figure 2(a)). Reaction temperature is another important factor for RT-LAMP amplification, which impacts not only the activity of the enzymes but also the hybridization capability and the efficiency between the primers and the target [39]. We optimized different reaction temperatures ranging from 50.6°C to 68.6°C for the RT-LAMP reaction and found out the amplification reached its best performance when the temperatures were 60.3°C and 62.6°C, respectively (Figure 2(b)). Considering the activity of RNA reverse transcriptase, we finally set the temperature on the instrument as 60°C for the amplification and detection.

### 2.3. Analytical Sensitivity of Real-Time LAMP

**Figure 3:**
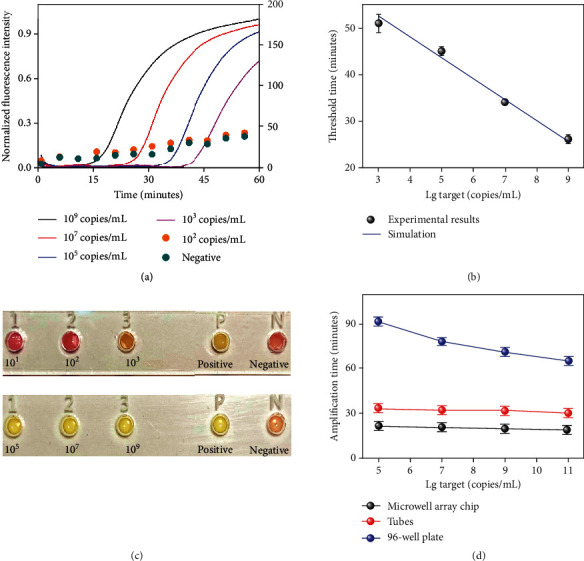
The performance of the RT-LAMP system for the detection of ORF1ab plasmids on a multiplexed microwell array and a real-time PCR instrument. (a) Real-time amplification curves with serially diluted targets ranging from 10^2^ copies/mL to 10^9^ copies/mL (1-4, normalized real-time amplification curves) and ddH_2_O as negative control: (1) 10^9^ copies/mL (black line); (2) 10^7^ copies/mL (red line); (3) 10^5^ copies/mL (blue line); (4) 10^3^ copies/mL (pink line); (5) 10^2^ copies/mL (orange circle); (6) negative control (cyan circle). (b) The threshold time as a function of the target concentration. The data was fitted with linear regression against the logarithm of the concentration. Error bars represent the standard deviation of 3 independent repeats. (c) Images of the multiplexed device taken after 25 min incubation for the detection of the virus at 10^9^, 10^7^, 10^5^, 10^3^, 10^2^, and 10^1^ copies/mL, respectively. The results show a detection limit of 10^3^ copies/mL for ORF1ab plasmids. (d) Comparison of reaction times of the RT-LAMP amplification in the microwell array chip (black line), in the tubes (red line), and in the 96-well plate (blue line) with serially diluted target ranging from 10^5^ copies/mL to 10^11^ copies/mL.

Various methods have been used for end-point analysis of the products from LAMP reactions, including gel electrophoresis, lateral flow immunoassay, turbidimetry, or pyrosequencing. However, these methods do not allow real-time detection and require further processing and instrumentation [40, 41]. Therefore, clinical trials of RT-LAMP reactions are commonly conducted on a PCR instrument because it can provide precise temperature control and real-time visualizing the amplification dynamics [42]. In this work, the sensitivity of the RT-LAMP assay was evaluated on both real-time PCR instrument and our portable instrument by using the first group of primers with different concentrations of ORF1ab gene plasmids ranging from 10^2^ copies/mL to 10^9^ copies/mL under 60°C. The results of real-time amplification performed on a PCR instrument indicate that the RT-LAMP system achieved the limit of detection (LOD) of 1,000 copies/mL for the SARS-CoV-2 within 40 min (Figure 3(a)). The corresponding *T*_*t*_ values of the RT-LAMP systems with triplicate measurements towards target concentrations from 10^9^ copies/mL to 10^3^ copies/mL are increased from 26 mins up to 51 mins (Figure 3(b)). Moreover, with the addition of different concentrations of plasmids into the microwells, a visible color was easily observed from pink to yellow, showing that the LOD of the portable detection system also achieves 1,000 copies/mL within 25 min from triplicate measurements (Figure S5).

Notably, the conical-shaped microwell demonstrated a faster read-out speed (~20 min) than commercial PCR tubes (>30 min) and the well of a 96-well plate (>60 min) on the instrument with a serially diluted target ranging from 10^5^ copies/mL to 10^11^ copies/mL (Figure 3(d)). To investigate the reason, we modelled and simulated the heat transfer efficiency of the unique conical shape and the commercial tubes for RT-LAMP reaction by using COMSOL Multiphysics (Figure S6). The quantitative results show that the unique conical shape enables a faster heat transfer efficiency, resulting in a faster reaction speed than commercial tubes (Figure S7).

### 2.4. Clinical Validation

**Figure 4:**
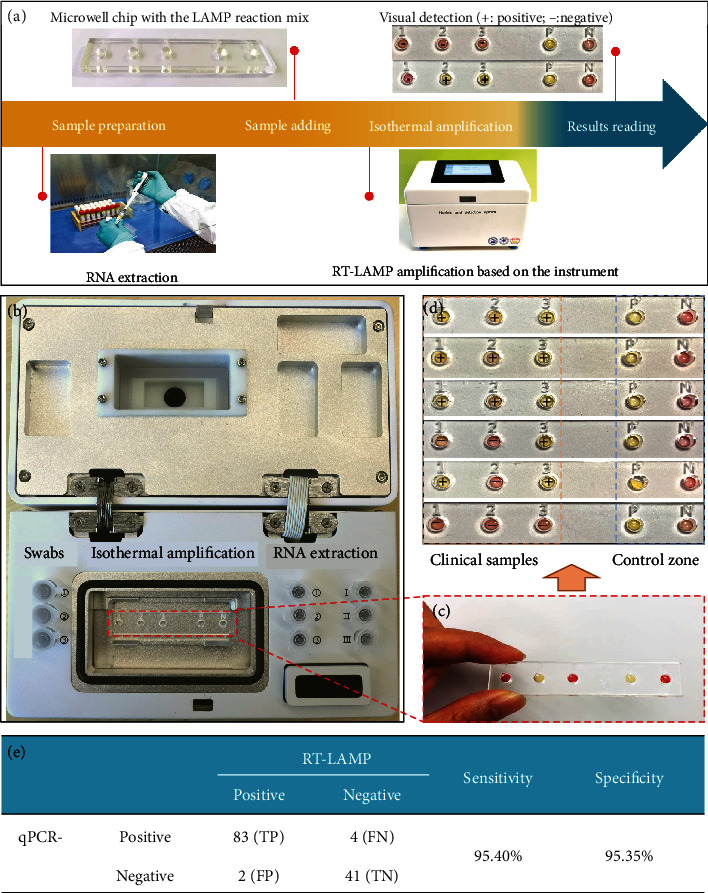
Clinical validation of the RT-LAMP detection platform with the real samples of COVID-19 patients from the West China Hospital, Chengdu. (a) The detection process using our platform, including RNA extraction, amplification, and results reading. (b) Photographs of the microwell chip mounted on the platform prior to the addition of RT-LAMP buffer and clinical samples. (c) The color in microwell changes from pink to yellow with positive samples. (d) Detection results of clinical samples on the microwell array. (e) The diagnostic accuracy for the clinical samples based on our platform against reference laboratory diagnosis (compared with RT-qPCR as a benchmark), based on a total of 130 clinical samples (87 COVID-19 positive and 43 negative).

To validate the proposed system, double-blind, randomized controlled clinical trials were conducted at the West China Hospital, Chengdu, China, with 130 clinical samples that have been diagnosed by the RT-qPCR method. 87 samples were confirmed with SARS-CoV-2 infection and 43 negatives according to the RT-qPCR system (Table S2). The clinical samples were detected through RNA extraction and isothermal amplification based on the portable instrument (Figures 4(a)–4(d)). Both sensitivity and specificity of the platform were assessed. The sensitivity refers to its ability to correctly identify the patients infected with SARS-CoV-2 while the specificity refers to the ability to correctly identify the specimen without infections. The test outcomes were defined as true positive (TP), false positive (FP), true negative (TN), and false negative (FN). This platform exhibited both high sensitivity and high specificity of 95.40% and 95.35%, respectively, in terms of the diagnosis results (Figure 4(e)).

## 3. Discussion

In this work, we implemented a portable and low-cost RT-LAMP platform for rapid diagnosis of COVID-19. The platform integrated full functions, including sample preservation, RNA extraction, thermal environment, and quantitative analysis. Containing a custom-made detection instrument and a low-cost microwell array biochip, it achieves high-throughput detection of SARS-CoV-2 RNAs from respiratory swab. We designed six novel primers for identifying the conserved regions of the ORF1ab gene of SARS-CoV-2. The RT-LAMP detection platform exhibited a low detection limit of 1,000 copies/mL with high specificity and high sensitivity, as validated with 130 clinical samples.

The total cost for fabricating the whole system was less than $600, including the detection instrument ($555.70) and microwell chip/primers ($1.57) (Table S3). For COVID-19 testing, the proposed portable instrument may find its suitability in some special scenarios, for example, customs and community hospitals, because of its advantages of low-cost (vs. PCR instruments) and rapid detection (25 min). In these scenarios, trained personnel would administer the test. We are currently developing a high throughput microwell chip that detects up to one hundred samples on one chip (Figure S8). Each microwell was marked on the left (row A-G) and above (column 1-14) to be easily addressed in testing procedure. Overall, the proposed platform offers both sensitivity and specificity that are comparable to RT-qPCR, yet with significantly lower cost, especially ideal for the regions with limited central laboratories, skilled personnel, and resources.

## 4. Materials and Methods

### 4.1. Apparatus and Regents

All real-time RT-qPCR and RT-LAMP reactions were performed on a qPCR machine (Applied Biosystems® 7500, Thermo Fisher Scientific, UK). A single-sided MicroAmp® Optical Adhesive Film was purchased from Thermo Scientific. Polymethyl methacrylate (PMMA) plate was obtained from Wanglong Materials (Shenzhen, China). Three sets of LAMP primers were designed using LAMP primer software Primer Explorer V5 (http://primerexplorer.jp/e/intro/index.html; Eiken Chemical Co., Japan). For increasing the reliability of the results, we included the BRAC1 gene (see Table S2 for detailed sequences) as the positive control, which resulted in less possibilities of false-negative results [31]. All primers and plasmids were synthesized by Sangon Company (Shanghai, China). Extraction of RNA was based on a nucleic acid extraction kit (3DBiopharm, 20200219, China) and a nucleic acid isolation machine (3DBiopharm, NP968-C, China). All clinical samples were provided and tested at the West China Hospital, Chengdu, under the authorization of the Chinese CDC. The research on the multiplexed and point-of-care diagnosis of COVID-19 using clinical samples had been approved by the ethical committee at the West China Hospital, Chengdu, China (No. 2020 (267)).

### 4.2. Design and Fabrication of the Portable Instrument

The hardware block schematic of the custom-made device for point-of-care SARS-CoV-2 detection is shown in Figure S1. At the core of our system, we used an ARM STM32 microcontroller that could be programmed on-board through an in-circuit serial programming interface. The temperature sensor is designed to perceive the temperature of the instrument, whose specific values will be shown on the liquid crystal display. Once the temperature deviates from the set point, the corresponding drive circuit will be triggered to either heat up or cool down, thus maintaining a constant temperature. The liquid crystal display, with all sets, can realize the friendly man-machine interaction function. A digital camera and an algorithm for color intensity analysis were designed to accompany the portable instrument and to provide a user-friendly interface for data display on the LCD (Figure S2). During detection, the user will firstly turn on the instrument with a home page and a process of self-infection before work (Figure S2a). Subsequently, it will display the real-time status of the instrument (Figure S2b). Some amplification setups, including temperature and time, could be set through touch control (Figure S2c). Finally, the instrument enables the functions of LAMP reaction and detection. The data and graphs will be shown on the screen (Figure S2d). Moreover, they could be stored on the device.

### 4.3. Fabrication of Multiplexed Microwell Chip

The microwell chip was fabricated by etching conical wells into a 5 mm thick PMMA plate using the high precise 40 W CO_2_ Laser Engraver Machine Cutter (HPC Laser, Ltd.). The protocol utilizes laser cut with conditions of power: 30%, frequency: 2500 Hz, resolution: 1200 dpi, and programmed linewidth: 10 *μ*m. The shapes of the conical wells, with the top diameter of 500 *μ*m and the bottom diameter of 3 mm, were laser-machined by programming (Figure S3). We designed a single-row prototype of 5 microwells, whose total fabrication time was within 30 seconds. Moreover, a high throughput device with 100 microwells for simultaneous detection of 100 samples in one run was fabricated. A single-sided adhesive acetate film was used to seal plastic plate to form enclosed chambers for hosting the LAMP reaction and preventing the liquid from evaporation.

### 4.4. Real-Time RT-LAMP Reaction

The real-time RT-LAMP reactions were performed with the addition of the well-established fluorescent DNA-intercalating dye, SYBR^@^ Green, which is a green fluorescent nucleic acid dye and becomes highly fluorescent when it binds to dsDNA [43]. More importantly, this dye was readily compatible with real-time PCR instruments which were equipped with a 488 nm laser or any visible light excitation with a wavelength in the region. The RT-LAMP reaction was carried out using the WarmStart™ LAMP 2x Master Mix (DNA and RNA) from New England Biolabs (NEB). Typically, a final volume of 25 *μ*L reaction mixture, which contained 12.5 *μ*L of 2x Master Mix, 3.4 *μ*L of primer mix, 0.5 *μ*L of SYBR^@^ Green I, 5 *μ*L of RNA target, and 3.6 *μ*L of ddH_2_O, was mixed and used. The reaction buffer was optimized according to a previous study [30]. The LAMP reaction was run for 45 min at 60°C on a real-time PCR machine. The internal negative control was performed with the same composition with positive control but using ddH_2_O as the template.

### 4.5. RNA Extraction from Clinical Samples

RNA was extracted from throat swabs and blood samples collected from the patients with COVID-19 virus infection during the epidemic at the West China Hospital, Chengdu, in 2020. The respiratory specimens collected were firstly heated at 56°C for 30 min for inactivation. Subsequently, 300 *μ*L of sample solutions were transferred into a nucleic acid extraction kit (3DBiopharm, 20200219, China), which was based on the method of magnetic beads in a biological safety cabinetry. After that, 20 *μ*L of nucleic acid lyase was added to the above mixture. Finally, RNA was extracted by using a nucleic acid isolation machine (3DBiopharm, NP968-C, China) in about 26 min. To avoid the interference of TE buffer to RT-LAMP reaction, RNA extracts were eluted twice by RNase-free and DNase water.

### 4.6. RT-LAMP Assay on the Microwell Array Chip

After the extracted RNAs are transferred into the microwell, the upper side of the microwell is sealed with a single-sided adhesive film, forming an enclosed chamber for the isothermal amplification reaction. The RT-LAMP reaction on the multiplexed device was performed in a final volume of 25 *μ*L, including 12.5 *μ*L of WarmStart Colorimetric Lamp 2x Master Mix, 3.4 *μ*L of primer mix, 0.5 *μ*L of WarmStart™ colorimetric dye from NEB, 5 *μ*L of RNA target, and 3.6 *μ*L of ddH_2_O. The concentrations of the primers are 0.16 *μ*M for the outer primers (B3 and F3), 0.8 *μ*M for the inner primers (BIP and FIP), and 0.4 *μ*M for the loop primers (LoopB and LoopF), respectively. RT-LAMP on the device was performed on the portable instrument at 60°C for 25 min. For detection of clinical samples, throat swab sampling, inactivation, and RNA extraction were performed based on corresponding functional zones integrated on the instrument. The extracted RNAs were transferred into the microwell chip and placed on the area labelled “Isothermal Amplification” on the instrument, which could complete the tests within 25 min.

### 4.7. Heat Transfer Simulation

To simulate the heat transfer efficiency, a multiphysics model was created on COMSOL Multiphysics software. The boundary conditions were provided as inputs to the model, and a convective heat flux form was selected with the following equation [44]:

(1) $$\begin{array}{c} q_{0}=h\left(T_{\text{ext}}-T\right) \end{array}$$

where *h* = 10 W/(m^2^·K), *T*_ext_ = 293.15 K, and *T* = 333.15 K. The detailed boundary conditions were listed as follows: the thermal capacity at constant pressure was set as 1,464 J/(kg·K) and 385 J/(kg·K) for PMMA chips and reaction buffers, respectively; the thermal conductivity was set as 0.1 W/(m·K), 0.6 W/(m·K), and 400 W/(m·K) for PMMA chips, hot plate, and reaction buffer, respectively. The tangent coefficient of thermal expansion was set as 85 (1/K) for PMMA chips. According to the simulation results, the cloud images of temperature changes at key time nodes, including 1 s, 10s, 100 s, and 500 s were extracted.

## Supplementary Materials

Supplementary Materials Figure S1: schematic diagram of signal-conditioning circuit. (a) Hardware block schematic of the custom-made device for SARS-CoV-2 detection; (b–f) signal-conditioning circuits for (b) Peltier drive, (c) temperature sensor, (d) electric fan, (e) buzzer, and (f) indicator light. VDD and VSS represent the positive and negative power supplies, respectively. Figure S2: the custom-made portable instrument for data and setup display. (a) The home page of the instrument after starting up; (b) real-time status of the instrument; (c) real-time display during LAMP amplification with the multiplexed microwell chip; (d) amplification results displayed on the liquid crystal display. Figure S3: the top view and side view of the microwell device. Figure S4: real-time RT-LAMP amplification curves using different sets of primers on a real-time PCR instrument for 40 min at 60°C. The cyan lines were performed with the first group of primers, pink lines with the second group of primers, and black lines with the third group of primers. The targets in three different concentrations, including 10^7^ copies/mL (low concentration, L1-L3), 10^8^ copies/mL (middle concentration, M1-M3), and 10^9^ copies/mL (high concentration, H1-H3), were recorded based on different sets of primers. Figure S5: the pictures of the color change in the microwells with different concentrations of ORF1ab plasmids indicate that the limit of detection of our platform achieves 1,000 copies/mL. Figure S6: simulation results of heat transfer efficiency of the unique conical shape and the tubes on the heating plate. Figure S7: quantitative data of heat transfer efficiency of the unique conical shape and the tubes on the heating plate. Figure S8: a high throughput microwell array chip with 100 microwells is applied for sample testing on the platform. (a) The high throughput chip was designed with the same dimension so that it fits the slot of the heating module on the platform; (b) the detection results are shown in the LCD display; (c) photographs of the array with 100 microwells. Table S1: LAMP primers designed for SARS-CoV-2. Table S2: the clinical samples provided by the West China Hospital. Table S3: the cost for materials and fabrication of the instrument and the microwell chip.
