# Supplementary material for: Low-Cost and Scalable Platform with Multiplexed Microwell Array Biochip for Rapid Diagnosis of COVID-19
Source: Research (Wash D C). 2021 Mar 12;2021:2813643. doi: 10.34133/2021/2813643 (PMC7982056; doi:10.34133/2021/2813643)
Supplement: Supplementary Materials — Figure S1: schematic diagram of signal-conditioning circuit. (a) Hardware block schematic of the custom-made device for SARS-CoV-2 detection; (b–f) signal-conditioning circuits for (b) Peltier drive, (c) temperature sensor, (d) electric fan, (e) buzzer, and (f) indicator light. VDD and VSS represent the positive and negative power supplies, respectively. Figure S2: the custom-made portable instrument for data and setup display. (a) The home page of the instrument after starting up; (b) real-time status of the instrument; (c) real-time display during LAMP amplification with the multiplexed microwell chip; (d) amplification results displayed on the liquid crystal display. Figure S3: the top view and side view of the microwell device. Figure S4: real-time RT-LAMP amplification curves using different sets of primers on a real-time PCR instrument for 40 min at 60°C. The cyan lines were performed with the first group of primers, pink lines with the second group of primers, and black lines with the third group of primers. The targets in three different concentrations, including 107 copies/mL (low concentration, L1-L3), 108 copies/mL (middle concentration, M1-M3), and 109 copies/mL (high concentration, H1-H3), were recorded based on different sets of primers. Figure S5: the pictures of the color change in the microwells with different concentrations of ORF1ab plasmids indicate that the limit of detection of our platform achieves 1,000 copies/mL. Figure S6: simulation results of heat transfer efficiency of the unique conical shape and the tubes on the heating plate. Figure S7: quantitative data of heat transfer efficiency of the unique conical shape and the tubes on the heating plate. Figure S8: a high throughput microwell array chip with 100 microwells is applied for sample testing on the platform. (a) The high throughput chip was designed with the same dimension so that it fits the slot of the heating module on the platform; (b) the detection results are shown in the LCD d [file 2813643.f1.docx]

**Supporting Information**

**Low-cost and Scalable Platform with Multiplexed Microwell Array Biochip for Rapid Diagnosis of COVID-19**

Yang Wang^1,†^, Kaiju Li^1,‡^, Gaolian Xu^1,§^, Chuan Chen^‡^, Guiqin Song^‡^, ZaiZai Dong^†^, Long Lin^†^, Yu Wang^‡^, Zhiyong Xu^||^, Mingxia Yu^¶^, Xinge Yu^#^, Binwu Ying^‡*^, Yubo Fan^†*^, Lingqian Chang^†*^, Jia Geng^‡*^

^†^ Beijing Advanced Innovation Center for Biomedical Engineering, Key Laboratory for Biomechanics and Mechanobiology, School of Biological Science and Medical Engineering, Beihang University, Beijing, 100083, P.R. China.

^‡^ Department of Laboratory Medicine, State Key Laboratory of Biotherapy and Cancer Center, West China Hospital, Sichuan University and Collaborative Innovation Center, Chengdu 610041, China.

^§^ Nano Biomedical Research Centre, School of Biomedical Engineering, Shanghai Jiao Tong University, Shanghai 200030, P. R. China

^||^ Wuhan Chain Medical Labs, Wuhan, Hubei 430011, China.

^¶^ Department of Clinical Laboratory, Zhongnan Hospital of Wuhan University, Wuhan, Hubei 430071, China.

^#^ Department of Biomedical Engineering, City University of Hong Kong, Hong Kong, China

^1^ These authors contribute equally to this work

*Corresponding Authors: Lingqian Chang (lingqianchang@buaa.edu.cn), Jia Geng ([geng.jia@scu.edu.cn](mailto:geng.jia@scu.edu.cn)), Binwu Ying (yingbinwu@scu.edu.cn), Yubo Fan (yubofan@buaa.edu.cn)

**Figure S1**. **Schematic diagram of signal-conditioning circuit.** (a) Hardware block schematic of the custom-made device for SARS-CoV-2 detection; b-f, Signal conditioning circuits for (b) Peltier Drive, (c) Temperature Sensor, (d) Electric Fan, (e) Buzzer and (f) Indicator Light. VDD and VSS represent the positive and negative power supplies, respectively.

**Figure S2**. **The custom-made POCT instrument for data and setup display**. (a) The home page of the instrument after starting up; (b) Real-time status of the POCT instrument; (c) Real-time display during LAMP amplification with the multiplexed microwell chip; (d) Amplification results displayed on the liquid crystal display.

**Figure S3**. **The top view and side view of the microwell device, including the dimensions**.

**Figure S4**. **Real-time RT-LAMP amplification curves using different sets of primers on a real-time PCR instrument for 40 min at 60 ℃.** The cyan lines were performed with the first group of primers, pink lines with the second group of primers and black lines with the third group of primers. The targets in three different concentrations, including 10^7^ copies/mL (Low concentration, L1-L3), 10^8^ copies/mL (Middle concentration, M1-M3) and 10^9^ copies/mL (High concentration, H1-H3) were recorded based on different sets of primers.

**Figure S5**. **The pictures of the color change in the microwells with different concentrations of ORF1ab plasmids indicate that the limit of detection of our platform achieve 1000 copies/mL**.

**Figure S6**. **Simulation results of heat transfer efficiency of the unique conical shape and the tubes on the heating plate**.

**Figure S7**. **Quantitative data of heat transfer efficiency of the unique conical shape and the tubes on the heating plate.**

**Figure S8**. **A high throughput microwell array chip with 100 microwells is applied for sample testing on the platform**. (a) The high throughput chip was designed with the same dimension so that it fits the slot of the heating module on the platform; (b) The detection results shown in the LCD display; (c) Photographs of the array with 100 microwells.

**Table S1. LAMP primers designed for SARS-CoV-2.**

**Table S2**. **The clinical samples provided by the West China Hospital.**

| Sample | Sample type | ORF1ab qPCR assay  (Ct Value) | RT-LAMP assay |
| --- | --- | --- | --- |
| 1 | throat swabs | 28.9 | positive |
| 2 | throat swabs | 29.1 | positive |
| 3 | throat swabs | 19.3 | positive |
| 4 | throat swabs | 26.6 | positive |
| 5 | throat swabs | 29.3 | positive |
| 6 | throat swabs | 28.3 | positive |
| 7 | throat swabs | 31.15 | positive |
| 8 | throat swabs | 32.3 | positive |
| 9 | throat swabs | 25.4 | positive |
| 10 | throat swabs | 32.1 | positive |
| 11 | throat swabs | 29.6 | positive |
| 12 | throat swabs | 39.3 | positive |
| 13 | throat swabs | 38.2 | positive |
| 14 | throat swabs | 36 | positive |
| 15 | throat swabs | 28.27 | positive |
| 16 | throat swabs | 32.5 | positive |
| 17 | throat swabs | 30.16 | positive |
| 18 | throat swabs | 30.4 | positive |
| 19 | throat swabs | 32.3 | positive |
| 20 | throat swabs | 21.6 | positive |
| 21 | throat swabs | 32.5 | positive |
| 22 | throat swabs | 36.48 | positive |
| 23 | throat swabs | negative | positive |
| 24 | throat swabs | 29.68 | positive |
| 25 | throat swabs | 33.1 | positive |
| 26 | throat swabs | 30.2 | positive |
| 27 | throat swabs | 34 | positive |
| 28 | throat swabs | 27 | positive |
| 29 | throat swabs | negative | positive |
| 30 | throat swabs | 15.7 | positive |
| 31 | throat swabs | 26 | positive |
| 32 | throat swabs | negative | negative |
| 33 | throat swabs | 30.4 | positive |
| 34 | throat swabs | 26.2 | positive |
| 35 | throat swabs | 31.5 | positive |
| 36 | throat swabs | 30.9 | positive |
| 37 | throat swabs | 25 | positive |
| 38 | throat swabs | 33.9 | positive |
| 39 | throat swabs | 25.3 | positive |
| 40 | throat swabs | 36.89 | positive |
| 41 | throat swabs | 29.1 | positive |
| 42 | throat swabs | 30.8 | positive |
| 43 | throat swabs | 34.5 | positive |
| 44 | throat swabs | negative | negative |
| 45 | throat swabs | 29.7 | positive |
| 46 | throat swabs | negative | negative |
| 47 | throat swabs | negative | negative |
| 48 | throat swabs | 23.43 | positive |
| 49 | throat swabs | 33.24 | positive |
| 50 | throat swabs | negative | negative |
| 51 | throat swabs | negative | negative |
| 52 | throat swabs | 33.77 | positive |
| 53 | throat swabs | 36.7 | positive |
| 54 | throat swabs | negative | negative |
| 55 | throat swabs | 33.94 | positive |
| 56 | throat swabs | 28.86 | positive |
| 57 | throat swabs | 31.25 | positive |
| 58 | throat swabs | 36.38 | positive |
| 59 | throat swabs | negative | negative |
| 60 | throat swabs | negative | negative |
| 61 | throat swabs | 27.88 | positive |
| 62 | throat swabs | 35.86 | positive |
| 63 | throat swabs | 36 | positive |
| 64 | throat swabs | 36.37 | positive |
| 65 | throat swabs | 36.98 | positive |
| 66 | throat swabs | 36.75 | positive |
| 67 | throat swabs | 32.45 | positive |
| 68 | throat swabs | 31.85 | positive |
| 69 | throat swabs | 27.06 | positive |
| 70 | throat swabs | negative | negative |
| 71 | throat swabs | 32.15 | positive |
| 72 | throat swabs | negative | negative |
| 73 | throat swabs | 35.67 | positive |
| 74 | throat swabs | 34.59 | positive |
| 75 | throat swabs | 31.26 | positive |
| 76 | throat swabs | 36.36 | negative |
| 77 | throat swabs | 35.16 | positive |
| 78 | throat swabs | 28.13 | positive |
| 79 | throat swabs | 29.9 | positive |
| 80 | throat swabs | 32.77 | positive |
| 81 | throat swabs | negative | negative |
| 82 | throat swabs | 33.9 | positive |
| 83 | throat swabs | 34.47 | positive |
| 84 | throat swabs | 34.18 | positive |
| 85 | throat swabs | 29.1 | negative |
| 86 | throat swabs | 31.78 | negative |
| 87 | throat swabs | 31.18 | negative |
| 88 | throat swabs | 32.95 | positive |
| 89 | throat swabs | 28.77 | positive |
| 90 | throat swabs | 35.15 | positive |
| 91 | throat swabs | 32.39 | positive |
| 92 | throat swabs | 30.82 | positive |
| 93 | throat swabs | 31.38 | positive |
| 94 | throat swabs | 34.71 | positive |
| 95 | throat swabs | 38.87 | positive |
| 96 | throat swabs | 37.79 | positive |
| 97 | throat swabs | 39.71 | positive |
| 98 | throat swabs | 27.75 | positive |
| 99 | throat swabs | 35.48 | positive |
| 100 | throat swabs | negative | negative |
| 101 | throat swabs | 33.39 | positive |
| 102 | throat swabs | negative | negative |
| 103 | throat swabs | 32.72 | positive |
| 104 | Blood | negative | negative |
| 105 | Blood | negative | negative |
| 106 | Blood | negative | negative |
| 107 | Blood | negative | negative |
| 108 | Blood | negative | negative |
| 109 | Blood | negative | negative |
| 110 | Blood | negative | negative |
| 111 | Blood | negative | negative |
| 112 | Blood | negative | negative |
| 113 | Blood | negative | negative |
| 114 | throat swabs | negative | negative |
| 115 | throat swabs | negative | negative |
| 116 | throat swabs | negative | negative |
| 117 | throat swabs | negative | negative |
| 118 | throat swabs | negative | negative |
| 119 | throat swabs | negative | negative |
| 120 | throat swabs | negative | negative |
| 121 | throat swabs | negative | negative |
| 122 | throat swabs | negative | negative |
| 123 | throat swabs | negative | negative |
| 124 | throat swabs | negative | negative |
| 125 | throat swabs | negative | negative |
| 126 | throat swabs | negative | negative |
| 127 | throat swabs | negative | negative |
| 128 | throat swabs | negative | negative |
| 129 | throat swabs | negative | negative |
| 130 | throat swabs | negative | negative |

**Table S3.** **The cost for materials and fabrication of the instrument and the microwell chip**.

|  | Detection Instrument | | Microwell Chip | |  |
| --- | --- | --- | --- | --- | --- |
| 1 | temperature control module | $92.50 | Microwell device | $0.15 |  |
| 2 | display and control system | $77.20 | Primers | $0.03 |  |
| 3 | electric control system | $231.60 | Reaction buffer | $1.39 |  |
| 4 | plastic enclosure | $154.40 |  |  |  |
| SUM |  | $555.70 |  | $1.57 |  |
